# Case report: Uncommon presentation of *Salmonella* Dublin infection as a large paravertebral abscess

**DOI:** 10.3389/fmed.2023.1276360

**Published:** 2023-11-21

**Authors:** Kang An, Zengxiang Wu, Cejun Zhong, Shuangqing Li

**Affiliations:** ^1^General Practice Ward/International Medical Center Ward, General Practice Medical Center, West China Hospital, Sichuan University, Chengdu, Sichuan, China; ^2^Center for Infectious Diseases, West China Hospital, Sichuan University, Chengdu, Sichuan, China

**Keywords:** *Salmonella* Dublin, paravertebral abscess, thoracolumbar spine, abscess drainage, case report

## Abstract

**Background:**

*Salmonella* Dublin is a zoonotic pathogen that is associated with invasive infections and high morbidity and mortality rates. Here we present the case of a 78-year-old man with a rare manifestation of a paravertebral abscess in the thoracolumbar spine caused by *Salmonella* Dublin.

**Case presentation:**

The patient had a history of spinal tuberculosis and poorly controlled diabetes. The abscess was successfully managed by drainage, and a 12-week course of moxifloxacin resulted in complete recovery. *Salmonella* Dublin was identified using culture-based serotyping. The patient resided in an environment where cattle farming is common; he consumed raw beef and unpasteurized milk, suggesting a potential source of infection.

**Discussion:**

Increasing the awareness of *Salmonella* Dublin as a potential cause of spinal abscesses is important, particularly in patients with structural spinal abnormalities. The timely initiation of appropriate antimicrobial therapy based on susceptibility testing is recommended. This case highlights the pathogenic potential of *Salmonella* Dublin and emphasizes the importance of effectively managing invasive *Salmonella* infections.

## 1 Introduction

*Salmonella* is a gram-negative facultative anaerobic bacteria, belonging to Enterobacteriaceae. *Salmonellae* are the most commonly isolated bacterial agents responsible for food-borne disease outbreaks (1–3). Food products of animal origin serve as the primary sources for the transmission of these pathogens. Specifically, poultry, pigs, and cattle, along with their associated products such as meat, eggs, and milk, are frequently identified as food sources leading to outbreaks of human salmonellosis (4, 5). Notably, eggs are widely recognized as a significant reservoir, consistently linked to sporadic cases and larger outbreaks of human salmonellosis (4). Nontyphoidal *Salmonella* (NTS) serovars are the most common causative agents of self-limiting gastroenteritis. Invasive NTS infections have a higher case fatality rate than non-invasive ones and can progress to meningitis, and bone and joint focal infections (6). However, this pathogen is commonly underestimated and overlooked in the clinical setting because of its unknown pathogenic processes.

The estimated global burden of invasive NTS infection was 535,000 cases (95% uncertainty interval: 409,000–705,000), with an age-standardized incidence rate of 7.5 (95% confidence interval [CI]: 5.7–10.0) cases per 100,000 person-years (7). Although sporadic cases have been reported worldwide, they are rare in mainland China (8). *Salmonella Enteritidis* and Typhimurium are the most common causative agents of invasive NTS infections, whereas *Salmonella enterica* serovars Dublin, Choleraesuis, Heidelberg, and Virchow are associated with higher invasiveness (9, 10). Certain populations, such as malnourished infants, older individuals, and those with sickle-cell disease, human immunodeficiency virus (HIV) infection, or recent malaria, are particularly vulnerable (1). However, the emergence of ceftriaxone-and nalidixic acid-resistant *Salmonella* Dublin strains poses challenges for antimicrobial stewardship, which is currently unclear (11).

Paravertebral abscesses are rare manifestations of *Salmonella* infections (8, 12). A definitive treatment approach and the duration of paravertebral abscesses caused by *Salmonella* have not yet been established. In this case report, we present the case of a patient who was diagnosed with a large abscess in the right paravertebral region of the thoracolumbar spine caused by *Salmonella* Dublin based on clinical manifestations and traditional bacterial culture methods. The abscess was successfully managed with drainage and the administration of appropriate antibacterial agents, which resulted in a favorable prognosis despite delayed treatment.

## 2 Case description

A 78-year-old man, residing at a temple in a Tibetan rural area, was admitted to the hospital because of recurrent lumbago that had persisted for 5 years. The patient had experienced aggravating symptoms over a 3-month period prior to hospital admission. The patient was diagnosed with type 2 diabetes in 2003. In July 2015, the patient underwent anterior debridement and bone grafting with anterior internal fixation to treat L1–2 vertebral tuberculosis. The operation showed partial bone defect of L1-2 vertebral bodies, L1/2 intervertebral space stenosis, and a caseous abscess in the soft tissue around L1-2 vertebral bodies. During the surgery, debridement was performed on the soft tissues around the L1-2 vertebral bodies, and bone grafting and internal fixation were performed on the L1-2 vertebral bodies. The patient received oral isoniazid (H), rifampin (R), ethambutol (E), and pyrazinamide (Z) [HREZ] chemotherapy after surgery. After 6 months, pyrazinamide was discontinued. Subsequently, he received a 12-month HRE dose of chemotherapy (6HREZ/12HRE). Due to spinal stenosis, he underwent percutaneous endoscopic interlaminar lumbar decompression and L4–5 discectomy in September 2016. Since July 2018, he had experienced persistent lumbago along with a palpable mass on the right side of the lumbar region, approximately at the level spanning from T10 to L2. The patient did not report a cough, sputum production, or fever. On October 11, 2018, he visited the hospital of the People’s Government of Tibet Autonomous Region Office in Chengdu where routine blood tests revealed elevated levels of procalcitonin and C-reactive protein, increased erythrocyte sedimentation rate (ESR) and white blood cell count, and a decreased hemoglobin level. Computed tomography (CT) of the chest and lumbar spine revealed partial bone defect of L1-2 vertebral bodies, L1/2 intervertebral space stenosis, no obvious abnormality of internal fixation, and lobulated low-density shadow of right paravertebral psoas muscle. The patient received isoniazid, rifampicin, ethambutol, and levofloxacin as anti-tuberculosis treatment, but the back mass continued to grow in size. According to the patient’s description, the size of the mass gradually increased from 10 × 20 cm to 20 × 35 cm.

**Figure 1:**
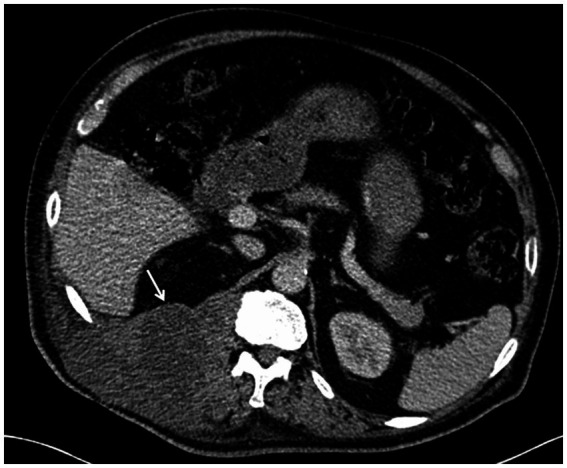
Contrast-enhanced computed tomography (CT) scans of the mass at the level of thoracic vertebrae 12 (T12). *Note:* The enhanced CT scan of the abdomen revealed multiple low-density masses in the right waist. The maximum cross-sectional area of the mass was approximately at the level of the T12, as shown in the figure.

**Figure 2:**
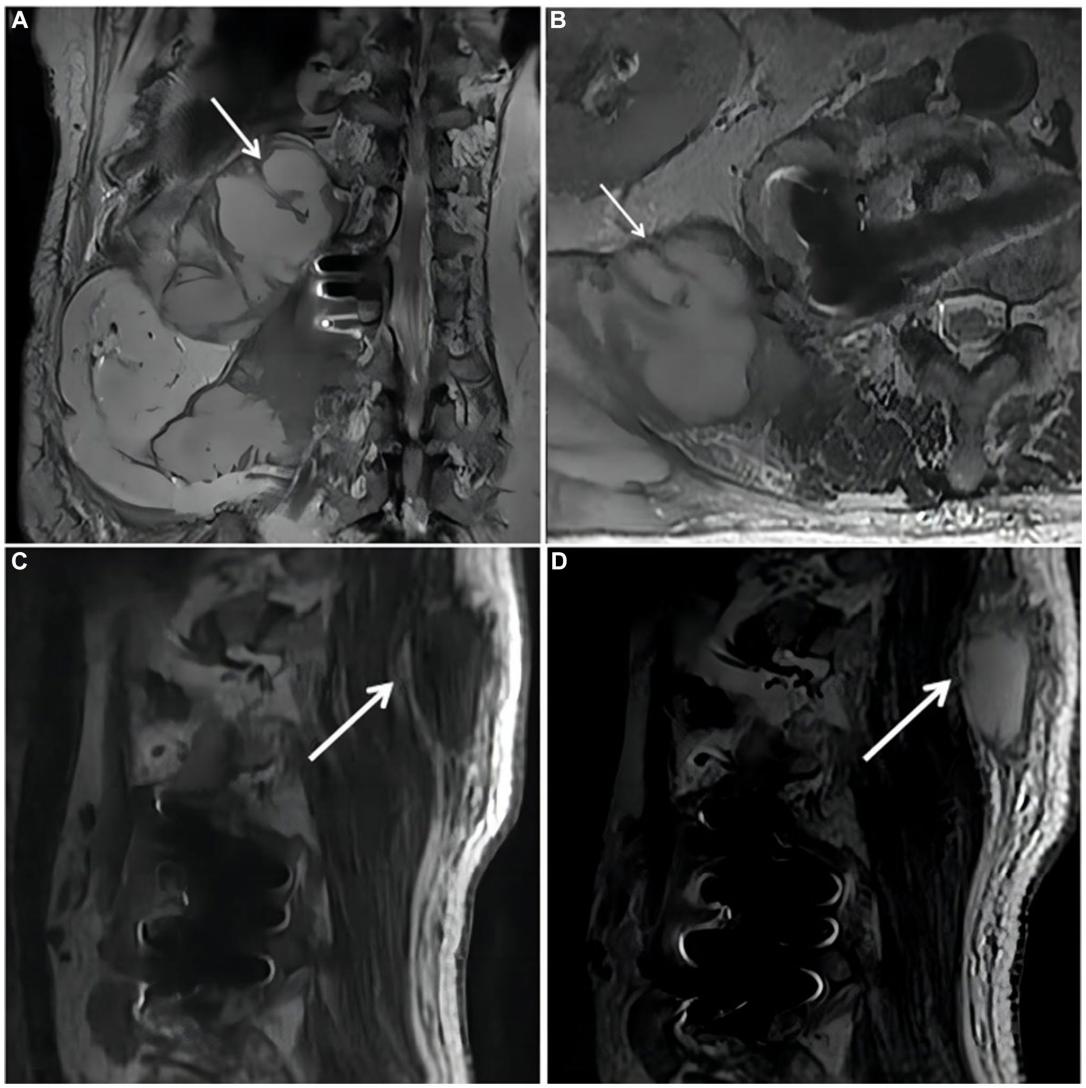
Magnetic resonance imaging (MRI) of the thoracolumbar spine. **(A)**. Coronal plane of the MRI scan revealed irregular patchy long T2 signal shadows at the right side of thoracolumbar vertebrae (starting from the level of T10). **(B)**. Cross section of the lumbar vertebra MRI scan revealed irregular patchy long T2 signal shadows at the right side (the level of L2). **(C,D)**. Sagittal plane of thoracolumbar spine MRI scan revealed long T1 and long T2 signal shadows, starting from the level of T10 and affecting subcutaneous tissue.

On October 18, 2018,the patient was admitted to our hospital for further evaluation and treatment after 1-week period of anti-tuberculosis treatment. Physical examination revealed tenderness in the thoracolumbar spine, specifically at the level spanning from thoracic vertebra 9 to lumbar vertebra 3. Additionally, a visible mass measuring approximately 20 × 35 cm was detected on the right side of the lumbar region. The test results show a hemoglobin level of 110 g/L, a fasting blood glucose of 8.44 mmol/L, and no abnormalities in liver and kidney function. The results of the infection-related tests are shown in eTable 1. Eight sputum smears were examined for bacteria, fungi, and mycobacteria, and all smears showed no abnormalities. Furthermore, various tests yielded negative results, including urinalysis, stool examination, electrolyte levels, liver and kidney function tests, serologic markers for viral hepatitis, HIV antibody test, fluorescent detection of tuberculosis bacilli DNA, tuberculin skin test, gamma interferon release assay, TORCH-IgM testing (antibody detection for toxoplasmosis, rubella virus, cytomegalovirus [CMV], and herpes simplex virus), Epstein–Barr (EB) virus DNA, CMV DNA, 1,3-β-D-glucan assay, and galactomannan antigen detection assay. An enhanced CT scan of the entire abdomen revealed significant swelling and multiple low-density masses within the right erector spinae, psoas major, and quadratus lumborum muscles. This roughly corresponds to the level between thoracic vertebra 10 and lumbar vertebra 4. The largest mass measured approximately 12.2 × 10.1 cm and displayed mild ring enhancement following CT scan contrast medium injection (contrast medium: iodixanol injection) (Figure 1). Magnetic resonance imaging (MRI) of the thoracolumbar spine revealed multiple irregular nodular and patchy lesions on the right side, spanning from the level between thoracic vertebra 10 and lumbar vertebra 3. These lesions exhibited long T1 and T2 signal intensities and affected the right psoas major, quadratus lumborum, and erector spinae muscles and subcutaneous tissues. The largest lesion measured approximately 12.4 × 9.1 cm (Figure 2).

**Figure 3:**
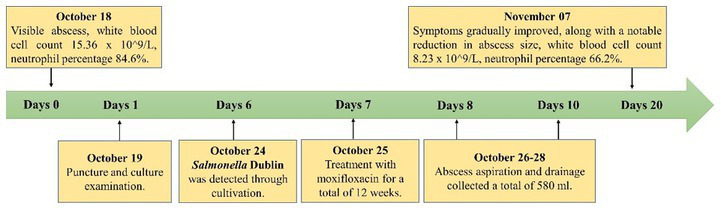
Timelines of patient’s the changes of infection indexes and treatment.

On October 19, 2018, purulent fluid (50 mL) was aspirated from two separate puncture sites. Microscopic examination of both samples yielded similar results, revealing a few gram-negative rod-shaped bacteria. The fluid appeared purulent and turbid. Owing to the sample viscosity, the microscopic evaluation revealed numerous nucleated cells (++++), moderate red blood cells (++), a few pus cells (+), and no epithelial cells. Blood agar plates were used for culturing *Salmonella*, and the VITEK® MS fully automated rapid microbial mass spectrometry detection system was employed for species identification. Serotyping was performed according to the White-Kauffmann-Le Minor scheme. On October 24, 2018, the culture results indicated the presence of *Salmonella* Dublin (cultured for 4 days and 16 h), with susceptibility testing revealing sensitivity to moxifloxacin. Susceptibility results were consistent across both culture samples. From October 25, 2018, the patient was given Moxifloxacin 0.4 g orally every day. Two separate attempts were made to culture and identify *Mycobacterium tuberculosis* from the fluid over a period of 6 weeks, but no tuberculosis bacilli were isolated. Detailed drug susceptibility results are provided in eTable 2 the Supplement. During hospitalization, multiple local abscess aspirations were performed at the bedside to relieve pressure, resulting in the gradual alleviation of symptoms and a significant reduction in abscess size. The drain was removed when the drainage flow rate reached <50 mL/24 h. The total volume of purulent fluid was 580 mL. Details regarding the changes of infection indexes and treatment are shown in eTable 1; Figure 3.

## 3 Diagnostic assessment: follow-up and outcomes

**Figure 4:**
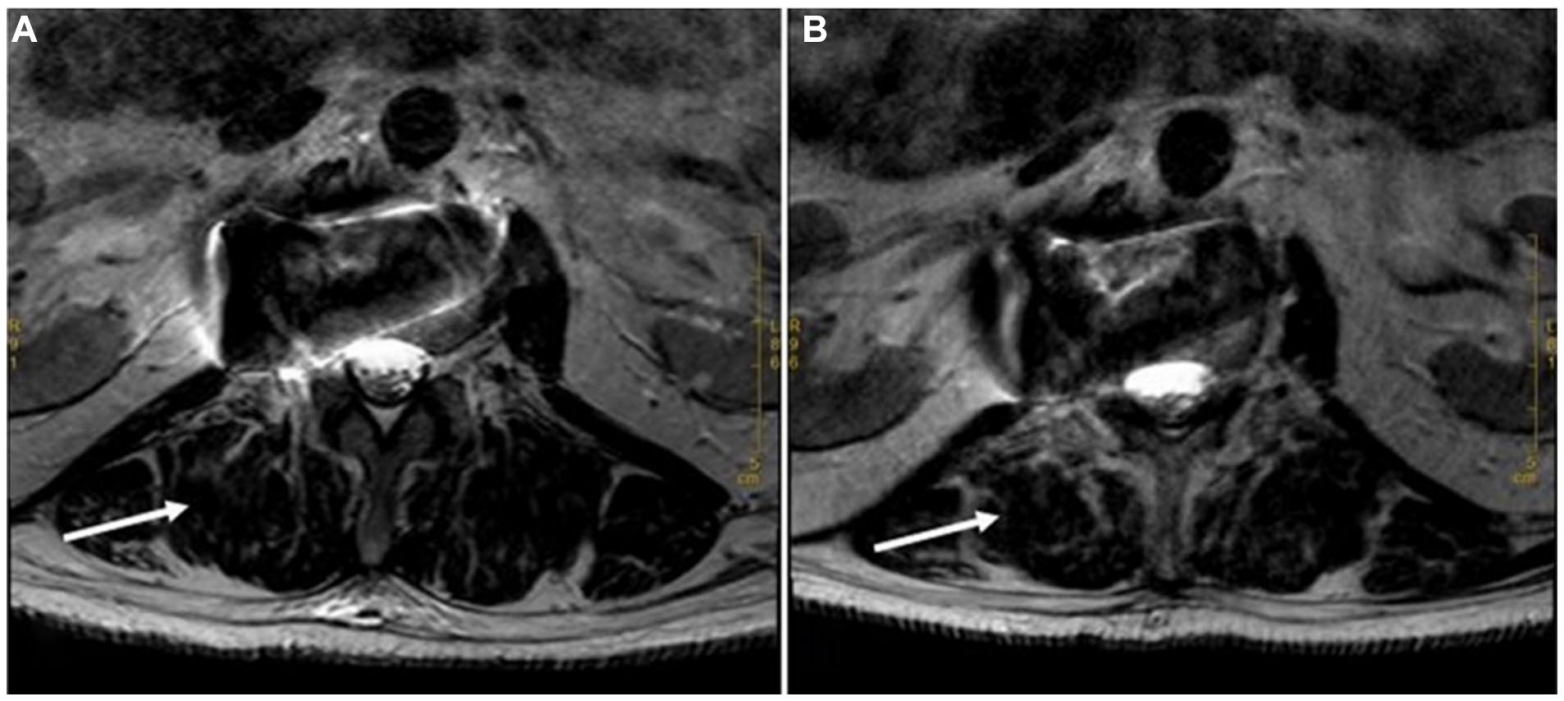
Long T2 MRI sequences at 6 Months and 18 Months after Discharge. **(A)**. Cross section of the lumbar vertebra MRI scan (March 2019) revealed flaky and strip-shaped shadows at the right side (the level of L2). It showed that the original shadow had been basically relieved. **(B)**. Cross section of the lumbar vertebra MRI scan (February 2020) showed no recurrence of the mass (the level of L2).

During the 4-year follow-up period, the patient consistently adhered to the prescribed treatment regimen of a 12-week course of oral moxifloxacin (400 mg once daily), leading to the gradual disappearance of lumbar pain symptoms. Follow-up MRI was performed in March 2019 and February 2020, as shown in (Figure 4). Subsequently, follow-ups were continued over an additional 2-year period, during which no recurrence of the disease was observed.

## 4 Discussion

*Salmonella* paravertebral abscess is a rare extra-intestinal manifestation of *Salmonella* infection. A review of over 7,000 cases revealed that only 1.7% of patients with *Salmonella* infections developed abscesses primarily affecting the gastrointestinal tract as periproctal, perineal, subphrenic, or appendiceal abscesses (12). Most patients had no fever or low fever. However, its exact pathogenesis remains unclear (13). The patient in our study was diagnosed with a localized infection caused by *Salmonella* Dublin, which was identified in pus cultures. The symptoms included a progressively enlarging abscess on the back, with CT findings suggestive of a paravertebral abscess, consistent with the presentation of a localized infection.

*Salmonella* and *Mycobacterium* infections occur in endemic regions. Both infections are characterized by the invasion of the bony spine, paravertebral tissue, spondylodiscitis, and psoas muscle abscesses (14, 15). When investigating the potential source of infection, we discovered that the patient resided in an environment where cattle farming was commonplace; he consumed homemade air-dried raw beef and unpasteurized milk. Based on this information, we hypothesized that the patient acquired the *Salmonella* Dublin infection through the consumption of contaminated food. In this case, the transmission route was considered to be hematogenous dissemination via the intestinal mucosa, leading to settlement in the adjacent soft tissues of the spine. The destruction of the affected vertebrae by tuberculosis can disrupt blood flow, causing turbulence, obstruction, and stasis, which promote bacterial localization and impair host defense mechanisms. Regarding the absence of positive blood culture results, we speculate that the administration of oral levofloxacin before admission may have suppressed bacteremia.

*Salmonella* Dublin is a highly dynamic microorganism that can thrive in both aerobic and anaerobic culture environments and is characterized by the presence of a Vi capsular polysaccharide antigen (16). The White-Kauffmann-Le Minor scheme is widely acknowledged as the definitive method for *Salmonella* serotyping, particularly in resource-limited settings. Compared with genomic techniques, this traditional method is more cost-effective and easily scalable. However, it is labor-intensive and time-consuming, requiring a minimum of 3 days to identify a single isolate. In this case, the patient presented with a paravertebral tuberculous abscess; this and the possible presence of multiple pathogenic strains required caution. Metagenomic next-generation sequencing (mNGS) is the preferred diagnostic method. However, due to the limited availability of mNGS in 2018 and the associated financial burden on the patient, mNGS was not used in this case (17).

Managing *Salmonella* can be challenging, particularly in the presence of necrotic tissue or implanted materials. Currently, there is no consensus on the treatment of invasive *Salmonella* infections. The treatment options described in previous case reports include drainage, debridement, and antibiotic therapy (18–20). In this case, the patient underwent drainage and antimicrobial treatment. Surgery is a promising and timely treatment option.

With the increasing prevalence of *Salmonella* resistance, third generation cephalosporins or fluoroquinolones may serve as effective alternative therapies for *Salmonella* Dublin infections (11). However, the specific choice of antimicrobial agent should be based on the results of susceptibility tests. The optimal duration of antibiotic treatment for localized infections remains uncertain; however, previous medical records suggest a course of at least 6–8 weeks (11). Depending on factors, such as the site of infection, extent of surgical debridement, presence of prosthetic materials (grafts, artificial joints, screws, plates, valves, or other implants), presence of residual fluid or necrotic tissue, immune status, and patient age, clinicians may consider extending the duration of antibiotic therapy (6–12 weeks) (21). No specific laboratory parameters were found to be helpful in monitoring the treatment response. We evaluated the treatment response by assessing the clinical resolution of pain, monitoring serial ESR, and examining radiological evidence of fusion, as in previous studies. The patient received moxifloxacin treatment for 12 weeks, which resulted in complete recovery upon assessment. This finding may aid in determining the appropriate treatment duration for patients with similar conditions.

Owing to the potential risk of *Salmonella* Dublin infection from the consumption of contaminated raw milk and beef, it is crucial for the government to engage in health promotion campaigns and enhance surveillance measures for individuals involved in livestock farming, cattle, and abattoirs. These initiatives should aim to prevent the spread of the disease through precise interventions (22). Appropriate antibiotic use should be practiced in both clinical settings and animal farming (23). It has been reported that the transmission of antibiotic-resistant *Salmonella* strains from animals to humans is associated with excessive antibiotic use in animal farming. Therefore, the continuous monitoring of *Salmonella* antibiotic resistance patterns is essential.

In conclusion, spinal abscesses caused by *Salmonella* Dublin infection are rare. Despite increased disease severity and treatment challenges, appropriate antimicrobial agents can effectively treat *Salmonella* Dublin infections. This case highlights the pathogenic potential of *Salmonella* Dublin in causing localized abscesses. When dealing with patients with spinal abscesses and spinal structural abnormalities, especially those who do not respond to anti-tuberculosis drugs, *Salmonella* Dublin infection should be considered. Selecting antimicrobial therapy based on bacterial susceptibility tests and the timely initiation of treatment are recommended.

Patient Perspective: The abscess drainage played a crucial role in my journey to full recovery. The healthcare professionals carefully explained the treatment plan, its expected duration, and potential side effects. This clarity was reassuring and helped me manage my expectations. During the 12-week course of Moxifloxacin, I appreciated the regular follow-up appointments to monitor my progress. The team’s willingness to answer my questions and address my concerns made a significant difference. It made me feel actively involved in my own healthcare decisions, and I believe it contributed to the successful outcome.

## Data availability statement

The raw data supporting the conclusions of this article will be made available by the authors, without undue reservation.

## Ethics statement

The studies involving humans were approved by the Ethics Committee on Biomedical Research, West China Hospital, Sichuan University. The studies were conducted in accordance with the local legislation and institutional requirements. The participants provided their written informed consent to participate in this study. Written informed consent was obtained from the individual(s) for the publication of any potentially identifiable images or data included in this article.

## Author contributions

KA: Data curation, Formal analysis, Software, Supervision, Writing – original draft. ZW: Investigation, Methodology, Writing – original draft. CZ: Data curation, Supervision, Validation, Writing – review & editing. SL: Conceptualization, Project administration, Resources, Visualization, Writing – review & editing.
