# Supplementary material for: Case report: Uncommon presentation of Salmonella Dublin infection as a large paravertebral abscess
Source: Front Med (Lausanne). 2023 Nov 21;10:1276360. doi: 10.3389/fmed.2023.1276360 (PMC10702734; doi:10.3389/fmed.2023.1276360)
Supplement: Supplementary file 1 [file Table_1.DOCX]

# eTable 1. Changes in Infection-Related Test Results

| Category | Outcome | | | | | | | | System of Units | Reference Value |
| --- | --- | --- | --- | --- | --- | --- | --- | --- | --- | --- |
|  | 2018.10.18 | | 2018.10.22 | | 2018.10.25 | | 2018.10.30 | 2018.11.07 |  |  |
| WBC | 15.36 | 10.72 | | 12.25 | | 10.28 | | 8.23 | 10^9^/L | 3.5-9.5 |
| NEUT | 12.99 | 9.48 | | 10.83 | | 7.73 | | 5.45 | 10^9^/L | 1.8-6.3 |
| NEUT% | 84.6 | 88.4 | | 88.4 | | 75.2 | | 66.2 | % | 40-75 |
| CRP | - | - | | 70 | | 33.6 | | 19.3 | mg/L | <5 |
| ESR | - | - | | 111.0 | | 100.0 | | 99.0 | mm/h | <43 |
| PCT | - | 0.16 | | 0.06 | | - | | 0.04 | ng/ml | ＜0.046 |

***Note:*** ESR: erythrocyte sedimentation rate, WBC: white blood cell, NEUT: neutrophil, PCT: procalcitonin, CRP: C-reactive protein.

**eTable 2. Detailed drug susceptibility results**

| **Antibiotic** | **Outcome** | **Sensitivity** | **Method** | **Antibiotic** | **Outcome** | **Sensitivity** | **Method** |
| --- | --- | --- | --- | --- | --- | --- | --- |
| Ampicillin | ＞＝32 | R | MIC | Piperacillin | ＞＝128 | R | MIC |
| Ticarcillin | ＞＝128 | R | MIC | Ticarcillin/Clavulanic acid | ＞＝128 | R | MIC |
| Piperacillin/Tazobactam | 8 | S | MIC | Amoxicillin/Clavulanic acid | 16 | I | MIC |
| Ampicillin/Sulbactam | ＞＝32 | R | MIC | Cefoxitin | 32 | R | MIC |
| Cefazolin | 16 | R | MIC | Cefuroxime | 16 | R | MIC |
| Ceftizoxime | ＜＝1 | S | MIC | Ceftriaxone | ＜＝1 | S | MIC |
| Cefotaxime | ＜＝1 | S | MIC | Ceftazidime | ＜＝1 | S | MIC |
| Cefepime | ＜＝1 | S | MIC | Cefotetan | ＜＝4 | R | MIC |
| Cefpodoxime | 2 | S | MIC | Cefuroxime Axetil | 16 | R | MIC |
| Aztreonam | ＜＝1 | S | MIC | Ertapenem | ＜＝0.5 | S | MIC |
| Imipenem | ＜＝0.25 | S | MIC | Meropenem | ＜＝0.25 | S | MIC |
| Doripenem | ＜＝0.12 | S | MIC | Tobramycin | ＜＝1 | R | MIC |
| Gentamicin | ＜＝1 | R | MIC | Amikacin | ＜＝2 | R | MIC |
| Nalidixic acid | ＞＝32 | R | MIC | Moxifloxacin | 0.5 | S | MIC |
| Norfloxacin | 2 | S | MIC | Ciprofloxacin | ＜＝0.25 | S | MIC |
| Levofloxacin | 0.5 | I | MIC | Compound sulfamethoxazole | ＜＝1/19 | S | MIC |
| Nitrofurantoin | 64 | I | MIC | Tetracycline | 2 | S | MIC |
| Doxycycline | * | S | MIC | Minocycline | * | S | MIC |
| Tigecycline | ＜＝0.5 | S | MIC |  |  |  |  |

***Note:***MIC(minimum inhibitory concentration)
